# On the Role of MoSe_2_ in Promoting Persulfate Activation by Fe-Based Catalysts: Dual Redox Cycles and Performance and Mechanism of Efficient Phenol Degradation in Water

**DOI:** 10.3390/molecules30224466

**Published:** 2025-11-19

**Authors:** Yirong Ren, Hao Zhao, Zerui Lu, Zuoyan Chen

**Affiliations:** 1 Gansu Institute of Natural Energy, Lanzhou 730000, Chinazhaohao15616@163.com (H.Z.);; 2 School of Metallurgy and Materials, University of Birmingham, Edgbaston, Birmingham B15 2TT, UK

**Keywords:** metal-organic frameworks, molybdenum diselenide, persulfate, advanced oxidation processes, phenol degradation

## Abstract

The recalcitrance and biological toxicity of phenolic pollutants pose a serious threat to the safety of aquatic environments, and developing efficient and stable catalytic degradation technologies is a key research focus in the current environmental field. In this study, a composite material (MSN) of NH_2_-MIL-101(Fe) modified by MoSe_2_ nanosheets was constructed via a one-step composite strategy, aiming to address the bottlenecks of low Fe^3+^/Fe^2+^ cycling efficiency and iron ion leaching in traditional Fe-based MOFs when activating peroxymonosulfate (PMS). Characterization results showed that MoSe_2_ nanosheets were uniformly dispersed on the surface of NH_2_-MIL-101(Fe), and strong electronic interactions existed between them, which significantly optimized the electronic environment of active sites. MSN-3 exhibited excellent performance in activating PMS for phenol degradation: the degradation rate reached 90% within 30 min, with a *k* = 0.073 min^−1^, which was much higher than that of other systems. It also showed good structural stability and cyclic regeneration ability. Mechanistic studies confirmed that the core active species in the MSN-3/PMS system are ^1^O_2_, •SO_4_^−^ and •OH. The two-dimensional layered structure of MoSe_2_ can serve as an efficient electron transport bridge to promote Fe^3+^/Fe^2+^ cycling; amino modification further optimizes the electron density of Fe active centers. The two synergistically construct a dual redox cycle of Fe^3+^/Fe^2+^ and Mo^4+^/Mo^6+^, significantly enhancing PMS activation efficiency and ^1^O_2_ production. This study provides a new strategy for designing Fe-MOFs-based PMS activation catalysts and also offers technical support for the practical treatment of recalcitrant organic pollutants in water.

## 1. Introduction

Phenolic compounds, as important chemical raw materials and intermediates, are widely present in industrial wastewater from industries such as pharmaceuticals, petrochemicals, and plastic manufacturing [1,2,3]. Among them, due to the chemical stability of its benzene ring structure, phenol is not only difficult to degrade by traditional biological treatment processes but also can accumulate in organisms through the food chain, inducing toxic effects such as cell membrane damage, protein denaturation, and DNA strand breakage. Long-term exposure can cause irreversible damage to human organs including the central nervous system, liver, and kidneys [4,5,6,7]. Therefore, the development of phenol degradation technologies with high efficiency, economy, and environmental compatibility is of urgent practical need and important environmental significance for ensuring aquatic environment safety and public health.

Advanced oxidation processes (AOPs) mineralize refractory organic compounds by generating highly oxidizing reactive species (e.g., •OH, •SO_4_^−^), making them a research focus in the current water treatment field [8,9]. Among them, AOPs based on peroxymonosulfate (PMS) activation exhibit significant advantages in phenol degradation—this is attributed to PMS’s high stability, convenient transportation and storage, as well as the longer half-life and wider pH applicability of the generated •SO_4_^−^ [10,11]. The performance of catalysts is critical to determining PMS activation efficiency, and Fe-based metal–organic frameworks (Fe-MOFs) have become ideal carriers for PMS activation due to their tunable porous structure, ultra-high specific surface area, and abundant Fe active sites [12,13,14,15]. Among these, MIL-101(Fe) is the most widely used in this field because of its excellent hydrothermal stability and high Fe content [16]. However, traditional MIL-101(Fe) suffers from low Fe^3+^/Fe^2+^ redox cycling efficiency, which leads to insufficient PMS activation and limited catalytic activity [17,18]. Furthermore, the material is prone to framework collapse under strong acid (pH < 3) or strong alkali (pH > 9) conditions, accompanied by massive Fe ion leaching. This not only reduces catalyst stability but also may cause secondary pollution, seriously restricting its application in complex water matrices [19,20]. Therefore, breaking through the pH dependence and cyclic activity bottlenecks of Fe-MOFs via precise material structure design and constructing efficient Fe-MOFs-based composite catalysts has become a key scientific issue to be urgently addressed in the current field of environmental catalysis.

$${\mathrm{Fe}}^{2+}+{\mathrm{HSO}}_{5}^{-}\to {\mathrm{SO}}_{4}^{∙-}+{\mathrm{OH}}^{-}+{\mathrm{Fe}}^{3+},\;k=3\;\times {\;10}^{4}{\;M}^{-1}s^{-1}$$

To solve the above bottlenecks, researchers have proposed a dual-strategy regulation approach involving cocatalyst introduction and surface functional modification [21,22,23]. For cocatalyst selection, heterogeneous sulfides exhibit higher stability and sustainability than homogeneous reducers, owing to their excellent electron transfer capability [24,25]. Among these, two-dimensional transition metal molybdenum diselenide (MoSe_2_) shows unique advantages in promoting Fe^3+^/Fe^2+^ cycling: it has a distinctive Se-Mo-Se sandwich layered structure (with an interlayer spacing of ~0.65 nm), high-density active sites exposed at the edges, and higher electrical conductivity and electron transfer rate compared to MoS_2_ [26,27,28,29]. Meanwhile, MoSe_2_ maintains high stability in acidic media, has low environmental toxicity, and its preparation cost is controllable—these features further highlight its application potential as a cocatalyst [30,31]. In terms of surface functionalization, amino (-NH_2_) modification can simultaneously improve the adsorption performance and electron conduction efficiency of MOFs through proton-coupled electron transfer effects [32,33,34]. Liu et al. [35] confirmed through their research that -NH_2_ modification can significantly reduce the charge transfer resistance of MIL-101(Fe) and enhance its interfacial interaction with PMS. Based on this, by combining the electron transfer advantages of MoSe_2_ with the active site regulation effect of -NH_2_ modification, this synergistic structural design not only retains the inherent porous structure and Fe active centers of NH_2_-MIL-101(Fe) and maintains the high specific surface area characteristics of MoSe_2_ ultrathin nanosheets, but also constructs an abundant active site library and efficient charge transfer channels through tight interfacial contact between components, laying a key structural foundation for the efficient implementation of subsequent PMS activation and pollutant degradation reactions.

This study aims to construct a MoSe_2_/NH_2_-MIL-101(Fe) (abbreviated as MSN) composite material via a one-step composite strategy. With phenol as the target pollutant, it systematically investigates the material’s performance and mechanism in activating PMS for organic pollutant degradation. Firstly, characterization techniques including XRD, SEM, XPS, and FT-IR are used to analyze the crystal structure, morphological characteristics, surface chemical state, and inter-component interactions of the MSN composite. Secondly, the effects of key parameters (catalyst dosage, PMS concentration, initial pH, common inorganic anions, and cycling times) on degradation performance are investigated to determine the optimal reaction conditions and environmental adaptability of the system. Finally, combined with active species quenching experiments and electron spin resonance (ESR) characterization, the core active species in the system are identified, and the synergistic enhancement mechanism of MoSe_2_ and -NH_2_ modification is revealed. This study not only provides new insights for the design of efficient PMS activation catalysts but also offers technical references for the treatment of refractory phenolic pollutants in water.

## 2. Results and Discussion

### 2.1. Characterization of the Catalyst

**Figure 1:**
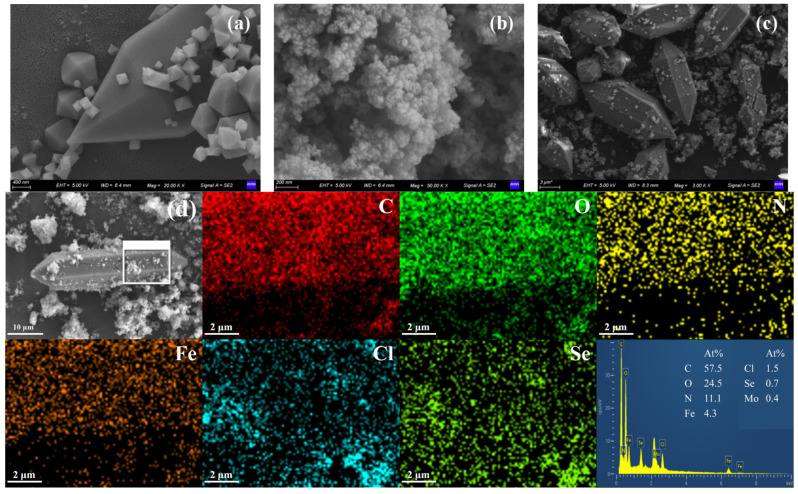
SEM image of NH2-MIL-101(Fe) (**a**); SEM image of MoSe2 (**b**); SEM image (**c**), EDX image (**d**), and EDS image (right side) of composite material MSN-3.

Combined SEM and EDS analyses systematically reveal the structural characteristics of the material from two dimensions: micromorphology and elemental distribution. As shown in Figure 1, NH_2_-MIL-101(Fe) exhibits a typical spindle-like morphology, and its regular geometric structure provides a natural porous framework for the reaction. In contrast, MoSe_2_ exists as a loose cluster morphology assembled from ultrathin nanosheets, creating structural conditions for the enhancement of catalytic activity. In the MSN-3 composite, MoSe_2_ ultrathin nanosheets closely adhere to and uniformly distribute on the surface of NH_2_-MIL-101(Fe) spindles, with no obvious agglomeration. The EDS mapping results further confirm that Mo and Se elements show uniform distribution throughout the composite, with no local enrichment observed. This directly verifies the effective compositing of MoSe_2_ and NH_2_-MIL-101(Fe).

**Figure 2:**
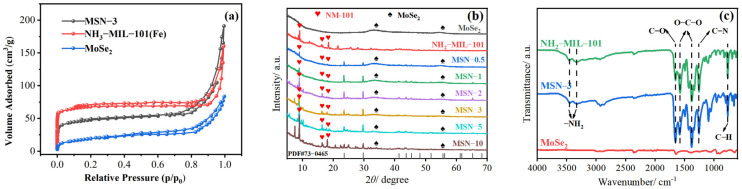
Adsorption–desorption isotherms of different samples (**a**); XRD patterns (**b**) and FT-IR spectra (**c**).

The results of the BET experiment are shown in Figure 2a. The adsorption–desorption isotherms of MoSe_2_, NH_2_-MIL-101(Fe), and the MSN-3 composite all conform to the characteristics of Type II isotherms, accompanied by H3-type hysteresis loops—indicating that all three materials have typical microporous structures. Among them, NH_2_-MIL-101(Fe) has a specific surface area of 307.26 m^2^/g, while the specific surface area of MoSe_2_ is only 71.92 m^2^/g. In contrast, the specific surface area of the MSN-3 composite decreases to 148.79 m^2^/g. It is inferred that this phenomenon originates from the fact that MoSe_2_ ultrathin nanosheets fill some of the microporous channels of NH_2_-MIL-101(Fe), causing slight pore blockage and thereby leading to a decrease in the specific surface area of the composite.

XRD analysis is used to verify the crystal structure integrity of the MSN-3 composite, with results shown in Figure 2b. Distinct characteristic diffraction peaks of MoSe_2_ clearly appear in the composite, corresponding to the (002), (100), and (110) crystal planes, with diffraction angles of 13.23°, 34.36°, and 55.62°, respectively. As the doping ratio of MoSe_2_ increases, the intensity of these characteristic peaks gradually enhances [28]. Meanwhile, no significant shift or attenuation occurs in the position and intensity of NH_2_-MIL-101(Fe)’s characteristic diffraction peaks, proving that the introduction of MoSe_2_ does not damage the framework crystal structure of NH_2_-MIL-101(Fe). Most notably, in composites with a low MoSe_2_ doping ratio, the characteristic diffraction peaks of MoSe_2_ disappear completely. This phenomenon implies that at low loadings, MoSe_2_ exists in the form of highly dispersed monolayer nanosheets rather than agglomerated crystal particles. This dispersion state maximizes the interfacial contact area between MoSe_2_ and NH_2_-MIL-101(Fe), providing a structural foundation for the exertion of their synergistic catalytic effect and the subsequent improvement of catalytic performance.

The FT-IR results are shown in Figure 2c. NH_2_-MIL-101(Fe) exhibited distinct absorption peaks at 765 cm^−1^, 1253 cm^−1^, 1380 cm^−1^, 1572 cm^−1^, 1652 cm^−1^, 3345 cm^−1^, and 3458 cm^−1^. Among these peaks, the absorption peak at 765 cm^−1^ is attributed to the vibration of Fe-O coordination bonds; the peaks at 1253 cm^−1^ and 1380 cm^−1^ correspond to the C-N stretching vibration and C-O-C symmetric stretching vibration, respectively; and the peak at 1572 cm^−1^ corresponds to the asymmetric stretching vibration of the O-C-O group in this MOF [36]. Notably, the characteristic peaks of both aforementioned materials appeared simultaneously in the infrared spectrum of the MSN-3 composite, which indicates that MoSe_2_ and NH_2_-MIL-101(Fe) have been successfully composited.

### 2.2. Effect of MoSe_2_ on the Degradation Performance of the Catalyst

**Figure 3:**
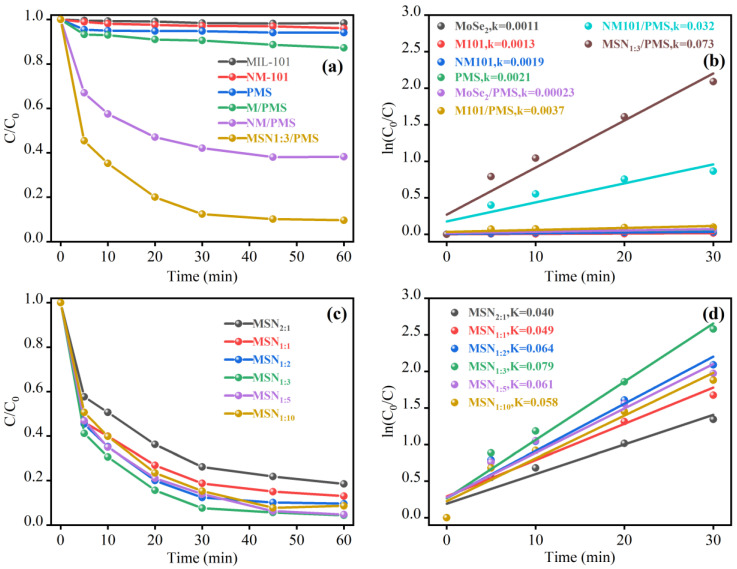
Degradation of phenol by different catalyst systems (**a**,**b**) and different doping ratios (**c**,**d**) of MoSe2/NH2-MIL-101(Fe).

Figure 3a systematically compared the differences in phenol degradation efficiency among various catalytic systems. Experiments showed that the degradation efficiency of phenol in pure component systems (MoSe_2_, M101, NM-101, PMS) and mixed systems (MoSe_2_/PMS, M101/PMS) was all below 10%. This result indicates that the self-decomposition of PMS can only generate a small amount of reactive free radicals, making it difficult to achieve efficient phenol degradation. Notably, the catalytic activity of the NM-101/PMS system was significantly enhanced: the phenol degradation rate reached 61.8% within 60 min, with a corresponding *k* = 0.032 min^−1^. This performance advantage stems from the electronic regulation mechanism of -NH_2_ on Fe active sites—the electron-donating property of -NH_2_ can increase the electron density of Fe^3+^ sites and reduce the energy barrier for the conversion of Fe^3+^ to Fe^2+^. As a key species for PMS activation, Fe^2+^ can efficiently trigger the decomposition of PMS to generate highly reactive •SO_4_^−^ and ^1^O_2_, thereby significantly improving PMS activation efficiency [37].

Among all tested systems, the MSN-3/PMS system exhibited the optimal catalytic performance: the phenol degradation rate reached 90% within 30 min, with a k value as high as 0.073 min^−1^, which was much higher than that of other systems. This significant advantage originates from the synergistic catalytic effect between MoSe_2_ and NM-101: the two-dimensional layered structure of MoSe_2_ can act as an efficient electron transport bridge, accelerating the electron transfer process from PMS to Fe^2+^, reducing the accumulation of Fe^2+^ after oxidation, and simultaneously promoting the continuous generation of reactive species such as •SO_4_^−^ and ^1^O_2_, ultimately achieving a significant improvement in catalytic efficiency [24].

The influence of MoSe_2_ loading on the catalytic performance of MSN-3 was further verified through experiments (Figure 3c). When the mass ratio of MoSe_2_ to NM-101 precursor increased from 1:10 to 1:3, the phenol degradation rate within 60 min gradually increased from 92% to 96%. This is because a moderate increase in MoSe_2_ can provide more active interfaces in contact with NM-101, enhance electron transfer efficiency, and does not damage the framework structure of NM-101 or the exposure of Fe active sites. However, when the loading ratio exceeded 1:3, the degradation efficiency showed a downward trend instead. The inferred reason is that excessive MoSe_2_ will agglomerate and cover the Fe active sites on the NM-101 surface, hindering the contact between phenol molecules and active sites; at the same time, the agglomerates increase the interface electron transfer resistance and weaken the synergistic catalytic effect between components.

**Figure 4:**
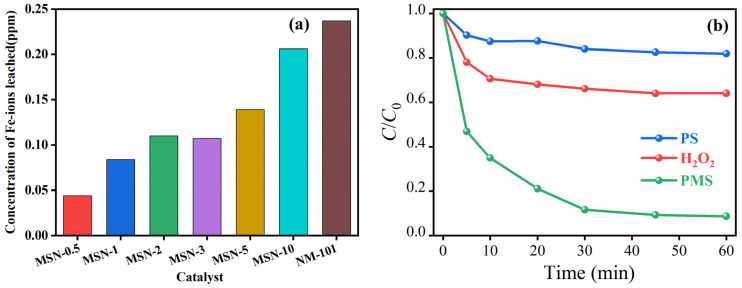
Iron ion leaching during the degradation of phenol at different doping ratios (**a**) and the degradation of phenol by different oxidants (**b**).

Figure 4a shows a systematic study on the structure-property relationship between iron ion leaching behavior and doping ratio in the MSN-3/PMS system. The experimental results indicate that the iron leaching rate in the MSN-3/PMS system is reduced by 58.7% compared to the NH_2_-MIL-101(Fe)/PMS system. This is primarily due to MoSe_2_ nanosheets effectively sealing surface defects and pores of NH_2_-MIL-101(Fe) through physical encapsulation and chemical bonding interactions, forming a more stable composite structure. Additionally, the two-dimensional conductive network of MoSe_2_ significantly promotes the rapid cycling of Fe^3+^/Fe^2+^, reducing the residence time of Fe^3+^ in the solution. This multi-stabilization strategy enables the catalyst to maintain excellent structural stability in a strong oxidizing environment, with the iron leaching concentrations consistently below 1.2 mg/L.

Figure 4b compares the performance differences in the MSN-3 catalyst in different oxidant systems (PMS/H_2_O_2_/PDS). The kinetic analysis of degradation shows that the catalytic efficiency of the PMS system is the highest (*k* = 0.073 min^−1^), which is 1.8 times and 4.1 times higher than that of the H_2_O_2_ (*k* = 0.041 min^−1^) and PDS (*k* = 0.018 min^−1^) systems, respectively. This significant difference stems from the unique asymmetric structure of PMS, which makes it easier to be activated by Fe^2+^, simultaneously generating two highly reactive species: •SO_4_^−^ (*E*_0_ = 2.5–3.1 V) and •OH (*E*_0_ = 2.8 V). Additionally, the introduction of MoSe_2_ extends the activation pathway of PMS from a purely radical mechanism (H_2_O_2_ system) to a synergistic mechanism involving both radical and non-radical (^1^O_2_) species. EPR testing confirms that the •SO_4_^−^ signal intensity in the PMS system is 3.5 times that of the PDS system. The symmetric structure and high bond energy (S-O = 140 kcal/mol) of PS result in a high activation energy barrier, while H_2_O_2_ is limited by the short lifetime (<1 μs) of •OH and its narrow pH adaptation range.

### 2.3. The Effect of Different Influencing Factors on the Degradation of Phenol by MSN-3

#### 2.3.1. Effect of Catalyst Dosage

**Figure 5:**
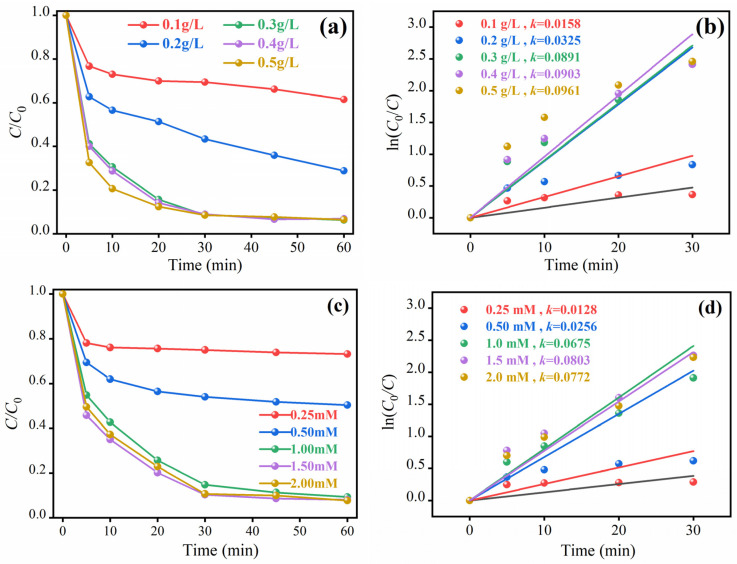
Effects of different catalyst dosages (**a**,**b**) and different PMS dosages (**c**,**d**) on the degradation rate of phenol.

To clarify the regulatory effect of MSN-3 dosage on the reaction system, this study systematically investigated the influence of MSN-3 dosage on phenol degradation performance, with the results shown in Figure 5a. Experiments revealed that when the catalyst dosage increased from 0.1 g/L to 0.3 g/L, the phenol degradation rate within 60 min significantly increased from 38.5% to 93.7%, and the corresponding k value increased synchronously, showing an obvious positive correlation. This change originated from the fact that the increased catalyst dosage directly provided more surface-active sites (Fe^2+^/Mo^4+^ active centers), which enhanced the adsorption and activation capacity for PMS, thereby accelerating the generation of reactive free radicals (•SO_4_^−^, ^1^O_2_). When the dosage exceeded 0.3 g/L, the increase in degradation efficiency tended to level off. This phenomenon occurred because the generation rate of reactive oxygen species reached a dynamic equilibrium, and PMS concentration became the rate-limiting factor of the reaction, which could not further increase with the continuous increase in catalyst dosage. In addition, excessive catalyst was prone to self-aggregation, leading to a decrease in effective specific surface area; some active sites were encapsulated and thus unable to participate in the reaction. Therefore, 0.3 g/L was adopted as the optimal catalyst dosage in subsequent experiments.

#### 2.3.2. Oxidant Dosage

The influence of PMS dosage on the phenol degradation kinetics of the MSN-3/PMS system is shown in Figure 5c. In the range of PMS concentration from 0.25 to 1.5 mM, the reaction activity was significantly enhanced with the increase in oxidant dosage: the phenol degradation rate within 60 min increased from 26.8% to 91.9%, and the k value rose from 0.0128 min^−1^ to 0.0803 min^−1^. This trend originated from the fact that PMS acts as a source of supply for •SO_4_^−^ and •OH—an increase in its concentration directly promotes the generation of more oxidative species, thereby enhancing the phenol degradation effect. However, when the PMS concentration exceeded 1.5 mM, the reaction kinetics underwent a turning point, and the improvement in degradation efficiency stagnated. This is mainly attributed to two reasons: first, excessive PMS triggers free radical self-quenching reactions, resulting in the effective free radical concentration not increasing synchronously with the PMS dosage; second, high-concentration PMS reduces the pH value of the solution, causing protonation on the surface of MSN-3 and weakening its ability to adsorb and activate PMS.

#### 2.3.3. Effect of pH

**Figure 6:**
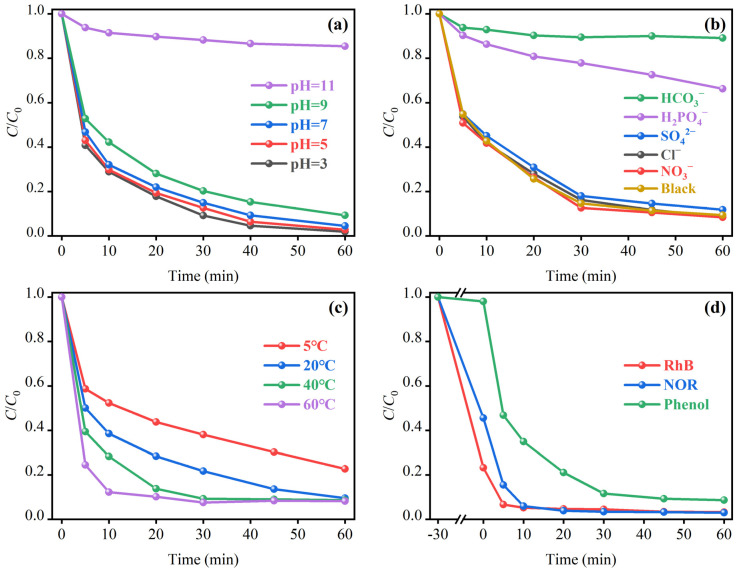
Effects of different conditions on the degradation rate of phenol: (**a**) different pH, (**b**) different inorganic anion systems, (**c**) different temperature systems, (**d**) different pollutants.

The aqueous pH value is a core parameter regulating the catalytic performance of the MSN-3/PMS system. On the one hand, it changes the existing form of PMS, thereby affecting the efficiency of PMS activation by the catalyst; on the other hand, it regulates the surface charge state of MSN-3 and alters the binding capacity between active sites, PMS, and pollutants. The dynamic change data of the system’s pH value during the reaction are detailed in Table S2 (Supplementary Materials, References [38,39,40,41,42,43] are cited in the Supplementary Materials). As shown in Figure 6a, the MSN-3/PMS system exhibited excellent wide pH adaptability: when the initial pH was in the range of 3–9, the phenol degradation rate remained above 90% within 60 min, and there was no obvious fluctuation in the degradation kinetic curve. However, when the initial pH was increased to 11, the catalytic performance of the system decreased sharply, with the phenol degradation rate being less than 20% within 60 min.

Further analysis of the mechanism was conducted based on the data in Table S2: When the initial pH = 11, the pH of the system rapidly decreased to 6.7~6.9 at the initial stage of the reaction due to the ionization of PMS (HSO_5_^−^ ⇌ H^+^ + SO_5_^2−^). During this process, the concentration of •SO_4_^−^ with a high oxidation potential decreases, while the concentration of •OH with a low oxidation potential increases, making •OH the dominant reactive species (Figure S4). As a result, the oxidation capacity of the MSN-3/PMS system is significantly weakened. Meanwhile, the strongly alkaline environment causes the Fe active sites in MSN-3 to detach from the framework and transform into catalytically inactive Fe(OH)_3_ precipitates. This process blocks the Fe^3+^/Fe^2+^ cycle and further inhibits the activation efficiency of PMS.

#### 2.3.4. Effects of Inorganic Anions

Inorganic anions commonly present in actual water bodies can affect the catalytic degradation process by altering the existing form of PMS, regulating solution pH, or quenching reactive species. To evaluate the anti-interference ability of the MSN-3/PMS system in complex water bodies, this study added 5 mmol/L of common anions to the reaction system, respectively, and investigated their effects on phenol degradation performance, with the results shown in Figure 6b. Experiments revealed that the addition of Cl^−^, NO_3_^−^, and SO_4_^2−^ had almost no impact on the system’s degradation efficiency, indicating that these three anions have extremely low reactivity with reactive species (•SO_4_^−^, ^1^O_2_). In contrast, the addition of H_2_PO_4_^−^ and HCO_3_^−^ significantly inhibited phenol degradation. For H_2_PO_4_^−^: on the one hand, it can react with •SO_4_^−^ and •OH to generate phosphate-related free radicals with extremely weak oxidizing ability; on the other hand, as a strong complexing agent, H_2_PO_4_^−^ can bind to the Fe^3+^/Mo^4+^ active sites on the MSN-3 surface, hindering the contact between PMS and active sites through steric hindrance effect and thus inhibiting PMS activation. For HCO_3_^−^: it is prone to hydrolysis in the solution, and the large amount of OH^−^ generated rapidly increases the solution pH. The strongly alkaline environment causes •SO_4_^−^ to convert to •OH with weaker oxidizability, and at the same time promotes the conversion of Fe active sites into non-catalytically active Fe(OH)_3_ precipitates, ultimately leading to a sharp decline in the system’s catalytic performance.

#### 2.3.5. Effect of Temperature

To clarify the regulatory effect of temperature on the catalytic kinetics of the MSN-3/PMS system, this study investigated the change in phenol degradation efficiency at different temperatures, with the results shown in Figure 6c. Experiments showed that increasing temperature significantly promoted the degradation reaction: at 5 °C, the phenol degradation rate within 60 min was 77.3%, while as the temperature rose to 60 °C, the degradation rate gradually increased to 99.2%, and the time for the reaction to reach equilibrium was significantly shortened. This is because increasing temperature reduces the reaction energy barrier between PMS and the active sites of MSN-3, improves the PMS activation rate, and promotes the generation of •SO_4_^−^ and ^1^O_2_; on the other hand, increasing temperature increases the molecular thermal motion rate in the reaction system, enhances the collision frequency between phenol molecules, PMS, and the active sites on the catalyst surface, and further accelerates the degradation reaction process.

#### 2.3.6. Material Suitability

To verify the universality of pollutant degradation by the MSN-3/PMS system, this study selected dye pollutants (rhodamine B, RhB) and antibiotic pollutants (norfloxacin, NOR) as target substances and investigated the system’s removal effect on different types of pollutants, with the results shown in Figure 6d. Experiments found that the MSN-3/PMS system exhibited excellent removal performance for all three pollutants, and possessed both efficient adsorption and catalytic degradation capabilities. For RhB, the adsorption capacity reached 76.8% within 30 min, and the total degradation rate reached 97.0% within 60 min; for NOR, the adsorption capacity reached 54.5% within 30 min, and the total degradation rate reached 97.4% within 60 min. This result indicates that MSN-3 not only has excellent PMS activation ability but also exhibits outstanding adsorption performance due to the abundant pore structure and polar groups (-NH_2_) on its surface. The adsorption process can enrich pollutants on the catalyst surface, further enhancing the subsequent catalytic degradation efficiency, which provides an application basis for its treatment of water bodies contaminated with multiple types of organic substances.

### 2.4. Study on the Cycling Stability of the Catalyst

The results of the cyclic experiment are shown in Figure S1a,b. MSN-3 still maintained excellent catalytic activity after 4 cycles of use: the phenol degradation rate within 60 min of each cycle was stably above 90%, and there was no obvious shift in the degradation kinetic curve; only the pseudo-first-order rate constant of the 4th cycle decreased slightly compared with the 1st cycle. This result indicates that the crystal structure and active sites of MSN-3 did not suffer significant damage during the cyclic process, and it has good structural stability and reusability, providing important performance support for subsequent practical applications.

**Figure 7:**
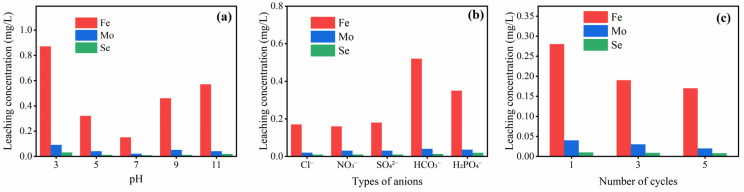
Ion leaching levels of MSN-3 under different reaction conditions: pH (**a**); inorganic anions (**b**); cycling experiments (**c**).

The leaching levels of Fe, Mo, and Se under different pH values, inorganic anion concentrations, and cycle periods are shown in Figure 7. Regarding pH values, the leaching concentrations of the three metals are the lowest under neutral conditions, where the material structure is the most complete, corresponding to the optimal catalytic activity. At pH = 3, the leaching concentration of Fe increased to 0.87 mg/L, and at pH = 11, it still reached 0.57 mg/L, while the leaching of Mo and Se fluctuated slightly. This indicates that strong acidic and alkaline environments will damage the structure of the material, leading to a large loss of Fe active sites and thus causing a decrease in activity.

Regarding inorganic anions, the leaching concentrations of the three metals are low in the presence of Cl^−^, NO_3_^−^, and SO_4_^2−^. However, the leaching of Fe increases significantly in the presence of HCO_3_^−^ and H_2_PO_4_^−^, while the leaching of Mo and Se shows no obvious fluctuation. This indicates that the pH-increasing effect from the hydrolysis of HCO_3_^−^ and the complexation of H_2_PO_4_^−^ will damage the material structure, affecting the structural integrity of the catalyst.

Regarding cycle periods, the leaching concentrations of Fe, Mo, and Se gradually decrease with the increase in the number of cycles. The leaching of Fe is the highest (0.28 mg/L) at the first cycle and decreases to 0.17 mg/L after 5 cycles, while Mo and Se return to their initial levels (Mo 0.02 mg/L, Se 0.008 mg/L). This reflects that a small number of unstable metal sites on the catalyst surface fall off during the first cycle, and the loss of active sites decreases as the cycles proceed, demonstrating excellent cycling stability.

### 2.5. Analysis of Intermediates and Toxicity

**Figure 8:**
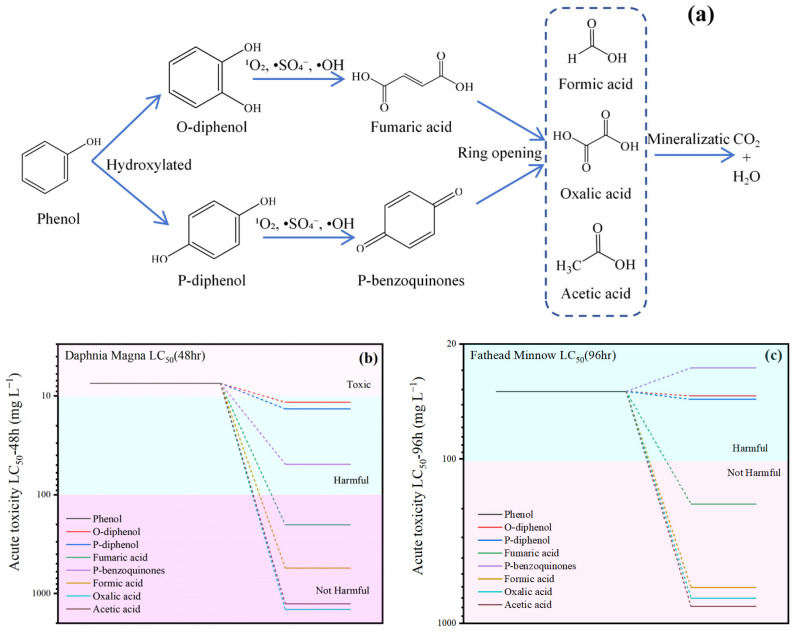
Intermediate product analysis (**a**); Toxicity analysis: Daphnia Magna LC_50_ (48 h) (**b**), Fathead Minnow LC_50_ (96 h) (**c**).

Through LC-MS analysis, the potential degradation pathway of phenol in the MSN-3/PMS system has been clarified (as shown in Figure 8): Phenol first undergoes hydroxylation to form o-diphenol and p-diphenol. Subsequently, p-diphenol is oxidized by reactive species in the system to p-benzoquinone, while o-diphenol generates fumaric acid through stepwise deacidification. Afterwards, the chemical bonds of fumaric acid and p-benzoquinone are further broken, converting into small-molecule organic acids such as oxalic acid, acetic acid, and formic acid, which are ultimately completely mineralized into carbon dioxide and water. The results of acute toxicity analysis show that the toxicity of most intermediates is lower than that of the parent phenol. Although the toxicity of some intermediates increases slightly, with the progress of the degradation process, the concentration of total organic pollutants in the system continues to decrease, so the overall toxicity of the solution shows a downward trend.

### 2.6. Identification of Active Free Radicals and Catalytic Mechanism

**Figure 9:**
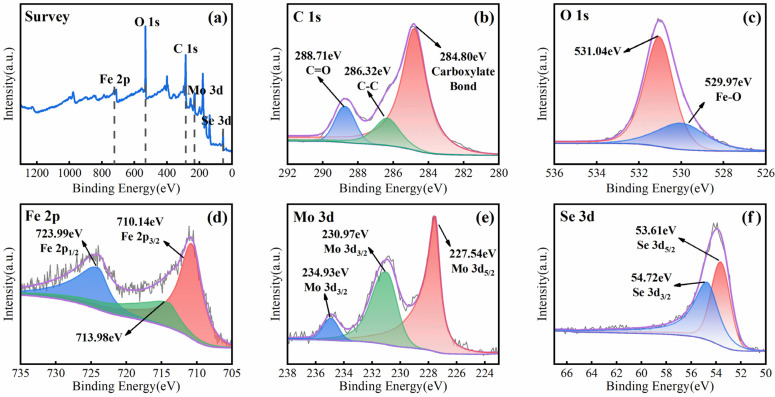
Full XPS spectrum (**a**) and XPS spectra of elements C 1s (**b**), O 1s (**c**), Fe 2p (**d**), Mo 3d (**e**), and Se 3d (**f**) of the MSN-3 sample.

The component synergy mechanism of MSN-3 was analyzed via X-ray photoelectron spectroscopy (XPS) from the perspectives of atomic valence states and electronic interactions, with the analysis results shown in Figure 9. The full-spectrum scan clearly shows the presence of six elements—Mo, Se, C, O, Fe, and N—in the material. As presented in Figure 9b, the high-resolution C 1s spectrum exhibits three characteristic peaks: 284.8 eV corresponds to carboxylate ester bonds in the benzoic acid ring, 286.32 eV is attributed to C-C single bonds, and 288.71 eV is the characteristic signal of C=O bonds in carboxyl groups [44]. In Figure 9c, the double peaks in the high-resolution O 1s spectrum at 529.97 eV (Fe-O) and 531.04 eV (chemisorbed oxygen) indicate the presence of metal–organic coordination bonds and surface hydroxyl groups, demonstrating that there are reactive oxygen sites on the composite surface that can participate in catalytic reactions [45]. The Fe 2p spectrum (Figure 9d) reveals the chemical state of iron: the main peaks at 710.14 eV (Fe^3+^ 2p_3_/_2_) and 723.99 eV (Fe^3+^ 2p_1_/_2_), along with the satellite peak at 713.98 eV, confirm the dominant presence of Fe(III) [46]. The Mo 3d spectrum shows characteristic double peaks of Mo^4+^ (227.54 eV and 230.97 eV) and a small amount of Mo^6+^ (234.93 eV), indicating partial oxidation of MoSe_2_ during the compositing process [47]. The double peaks at 53.61 eV and 54.72 eV in the Se 3d spectrum confirm the presence of Se^2−^. Its binding energy shifts by approximately 0.3 eV compared with that of pure MoSe_2_, suggesting strong electronic interactions between MoSe_2_ and NH_2_-MIL-101(Fe) [27]. In summary, XPS analysis clearly verifies the existence of a significant electronic coupling effect between the components of MSN-3. This electronic interaction can promote electron transfer at the Fe and Mo active sites and optimize the PMS activation pathway.

**Figure 10:**
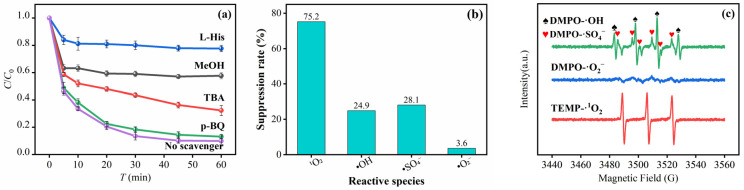
Quenching experiments of the MSN-3/PMS system (**a**); Inhibition rates of radicals and non-radicals (**b**); EPR spectra (**c**).

To clarify the core reactive species responsible for phenol degradation in the MSN-3/PMS system, this study investigated the contribution of different reactive oxygen species (ROS) through quenching experiments [48]. 1 M methanol (MeOH) was used to quench •SO_4_^−^ and •OH, 1 M tert-butanol (TBA) to quench •OH, 3 mM L-histidine (L-His) served as the specific quencher for ^1^O_2_, and 3 mM p-benzoquinone (p-BQ) was used to quench •O_2_^−^ [49,50]. The specific results are shown in Figure 10a. The pH change in the solution after adding only quenchers and phenol is shown in Figure S5. When p-BQ was added, the degradation rate only decreased from 93.42% to 86.9%, indicating that •O_2_^−^ had a minimal impact on the degradation process and only played an auxiliary role. When MeOH was added, the phenol degradation rate dropped from 93.5% to 42.4% within 60 min, demonstrating the presence of •SO_4_^−^ and •OH in the system. After adding TBA, the degradation rate decreased to 67.7%. Combining the selectivity differences in the two quenchers, it can be inferred that both •SO_4_^−^ and •OH were involved in the degradation process, and the contribution of •SO_4_^−^ was slightly higher than that of •OH. When L-His was added, the degradation rate sharply decreased to 22.3%. The relative contributions of each species were quantified by the inhibition rate of degradation efficiency, and the quantitative results are shown in Figure 10b. This significant inhibition effect confirmed that ^1^O_2_ was the core reactive species in the system, contributing the most to phenol degradation. In summary, the core reactive species for phenol degradation in the MSN-3/PMS system is ^1^O_2_, with •SO_4_^−^ and •OH participating synergistically, while •O_2_^−^ plays a weak role. This result provides a key basis for the subsequent construction of a catalytic mechanism model.

To further explore the reaction mechanism, this study directly captured the ROS generated in the MSN-3/PMS system via electron spin resonance (ESR) spectroscopy, with the experimental results shown in Figure 10c. Characteristic signals of various reactive species were clearly observed in the ESR spectra: typical tetrad peaks of DMPO-•OH adducts (peak intensity ratio 1:2:2:1) appeared in the system, and at the same time, the characteristic signal of DMPO-•SO_4_^−^ adducts was detected near these tetrad peaks—confirming that •SO_4_^−^ and •OH stably existed in the system and participated in the reaction. In addition, a weak signal of DMPO-•O_2_^−^ adducts was also observed in the spectrum, indicating that •O_2_^−^ was generated in small amounts during the reaction. Most crucially, the triple characteristic peaks (peak intensity ratio 1:1:1) of the TEMP-^1^O_2_ adduct showed an extremely strong signal with a symmetric peak shape, further confirming that ^1^O_2_ was the dominant reactive species in the system. This is completely consistent with the result of “significant inhibition of degradation by L-His” in the quenching experiment.

**Figure 11:**
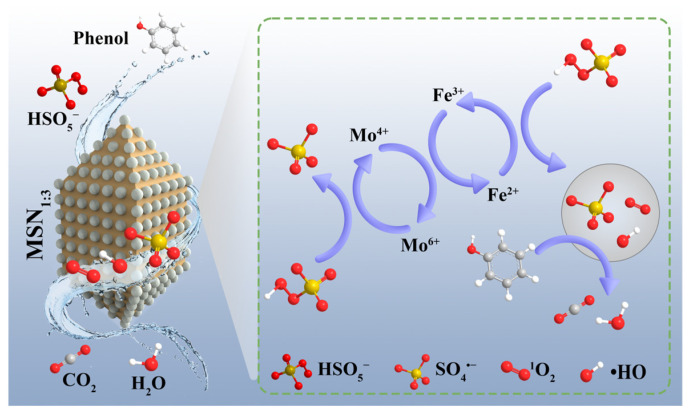
Mechanism diagram of phenol degradation by the MSN-3/PMS system.

Based on quenching experiments, ESR characterization, and analysis of the material’s electronic structure, a catalytic mechanism model for phenol degradation in the MSN-3/PMS system can be established (Figure 11). The core process is the generation of ^1^O_2_ in the heterogeneous persulfate catalytic system under the dual cyclic effect of Fe^3+^/Fe^2+^ and Mo^4+^/Mo^6+^. The generation of ^1^O_2_ may occur through different pathways. It has been reported that ^1^O_2_ in heterogeneous persulfate catalytic systems is mainly generated via the activation of PMS by Fe^2+^; in addition, dissolved oxygen may generate •O_2_^−^ in water, and •O_2_^−^ can also undergo conversion to ^1^O_2_ under the action of •OH and other species [51,52]. Meanwhile, due to the strong reducibility of the exposed Mo^4+^ in MoSe_2_ within MSN-3, it can reduce Fe^3+^ on the surface of NH_2_-MIL-101(Fe) to Fe^2+^, thereby promoting the Fe^3+^/Fe^2+^ cycle. The generated Fe^2+^ reacts rapidly with PMS, activating PMS to generate •SO_4_^−^, •OH, and •O_2_^−^; in turn, Mo^6+^ can be re-reduced to Mo^4+^ by capturing electrons in the system (e.g., electrons transferred by •O_2_^−^), forming the Mo^4+^/Mo^6+^ cycle. This dual cycle not only accelerates PMS activation but also avoids the accumulation of Fe^3+^ and the oxidative deactivation of Mo^4+^, significantly improving catalytic stability. Ultimately, ^1^O_2_, •SO_4_^−^, and •OH generated in the system jointly attack phenol molecules. By destroying the benzene ring structure, they gradually oxidize phenol to H_2_O and CO_2_, achieving the complete removal of the pollutant.

## 3. Experimental Section

### 3.1. Preparation of MSN Composite Materials

**Figure 12:**
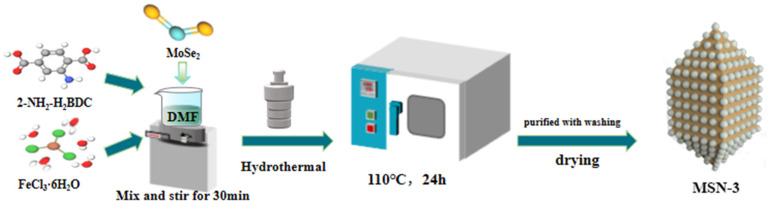
Flow chart of the preparation process of the MSN composite catalyst.

Preparation of Materials The preparation processes of MoSe_2_ and NH_2_-MIL-101(Fe) are described in Text S2 and Text S3, respectively. The preparation process of MoSe_2_/NH_2_-MIL-101(Fe) is similar to that of NH_2_-MIL-101(Fe), except that a certain amount of MoSe_2_ [with a mass ratio of m(MoSe_2_): m(FeCl_3_•6H_2_O) = 1:3] is added to the precursor for preparing NH_2_-MIL-101(Fe) before the reaction. The mixture is reacted at 110 °C for 24 h, and the black-brown MoSe_2_/NH_2_-MIL-101(Fe) (denoted as MSN-3) is obtained following the purification method of NH_2_-MIL-101(Fe). The preparation process is shown in Figure 12.

### 3.2. Catalytic Degradation Experiment

Experiments were conducted at a room temperature of (25 ± 2) °C. First, 200 mL of 20 mg/L phenol solution was added to a 250 mL beaker, and magnetic stirring (rotation speed: 500 rpm) was applied to homogenize the system. Subsequently, preset dosages of MSN-3 and PMS were added sequentially, and the start of stirring was recorded as the initial reaction time (t = 0). During the reaction, samples were collected at t = 0, 5, 10, 20, 30, 45, and 60 min, with 3 mL of the reaction solution transferred each time. After sampling, 0.1 mL of methanol was added to the sample to quench reactive species; after shaking uniformly, the mixture was filtered through a 0.22 μm aqueous microporous membrane to remove catalyst particles, and the filtrate was collected. Then, 2 mL of the filtrate was accurately transferred to a 25 mL colorimetric tube, and ultrapure water was added to bring the volume to the marked scale before determining the pollutant concentration. Each group of experiments was repeated 3 times to ensure data reliability.

### 3.3. Analytical Methods

The characterization methods of MSN-3 are provided in Text S5. Electron spin resonance (ESR) spectroscopy is used to directly capture the reactive oxygen species (ROS) generated in the MSN-3/PMS system: dimethylpyridine N-oxide (DMPO) is selected as the trapping agent for •SO_4_^−^, •OH, and •O_2_^−^, while 2,2,6,6-tetramethyl-1-piperidine (TEMP) serves as the specific trapping agent for ^1^O_2_.

## 4. Conclusions

In this study, through the synergistic design of MoSe_2_ and -NH_2_ modification, the significant electronic coupling effect between the components of MSN-3 was fully utilized. Specifically, Mo^4+^ in MoSe_2_ can promote the conversion of Fe^3+^ to Fe^2+^ in NH_2_-MIL-101(Fe) via electron transfer, while -NH_2_ modification enhances the adsorption and activation capacity of PMS by regulating the electron density of Fe active sites. Together, they synergistically construct the structural advantages of “abundant active sites and efficient charge transfer channels”. The MSN-3 composite was successfully prepared using a one-step composite strategy. Characterization results confirmed that the MoSe_2_ ultrathin nanosheets in MSN-3 closely and uniformly adhere to the surface of NH_2_-MIL-101(Fe) spindles, with no obvious agglomeration observed. Moreover, the introduction of MoSe_2_ did not damage the crystal framework integrity of NH_2_-MIL-101(Fe). In the MSN-3/PMS system, the phenol degradation rate reached 90% within 30 min, and the *k* (0.073 min^−1^) was much higher than that of other comparative systems. Furthermore, the MSN-3/PMS system exhibited excellent environmental adaptability, structural stability, and reusability. Quenching experiments and ESR spectroscopy confirmed that the core reactive species for phenol degradation in the MSN-3/PMS system was singlet oxygen (^1^O_2_). Its catalytic mechanism originates from the dual redox cycles of Fe^3+^/Fe^2+^ and Mo^4+^/Mo^6+^. This dual cycle not only accelerates the activation efficiency of PMS and avoids the accumulation of Fe^3+^ as well as the oxidative deactivation of Mo^4+^ but also promotes the conversion of •SO_4_^−^ and •OH to ^1^O_2_, ultimately achieving efficient phenol degradation. In summary, this study provides new insights for the structural design of high-efficiency PMS-activated catalysts.

## Data Availability

The original contributions presented in this study are included in the article.

## Supplementary Materials

The following supporting information can be downloaded at: https://www.mdpi.com/article/10.3390/molecules30224466/s1, Text S1: Experimental Materials; Text S2: Preparation of MoSe_2_; Text S3: Preparation of BNU-100 composite material; Text S4: Catalytic Degradation Experiment; Text S5: Analytical Methods; Table S1: Comparison of Removal Efficiency; Table S2: Changes in pH during the degradation of OFX at different initial pH values; Figure S1: Cycling stability experiments (a), (b) of the MSN-3/PMS system; Figure S2: pH variation trend; Figure S3: Post-reaction XRD pattern; Figure S4: EPR spectrum under pH = 11; Figure S5: Phenol removal rate (a); pH change in the solution (b) after reacting phenol with quenchers alone for 60 min.
