# Supplementary material for: On the Role of MoSe2 in Promoting Persulfate Activation by Fe-Based Catalysts: Dual Redox Cycles and Performance and Mechanism of Efficient Phenol Degradation in Water
Source: Molecules. 2025 Nov 19;30(22):4466. doi: 10.3390/molecules30224466 (PMC12654995; doi:10.3390/molecules30224466)
Supplement: Supplementary file 1 [file molecules-30-04466-s001.zip › molecules-3926070-supplementary.pdf]

Supplementary Material

On the role of $moSe_2$ in promoting persulfate activation by Fe-based catalysts: dual redox cycles and performance & mechanism of efficient phenol degradation in water

Yirong Ren[1,2], Hao Zhao[1], Zerui Lu[1], Zuoyan Chen[1,*]

[1] Gansu Institute of Natural Energy, Lanzhou, 730000, China; renyirong081466@hotmail.com (Y. R.); zhaohao15616@163.com (H. Z.); luzerui86@163.com (Z. L.)

[2] School of Metallurgy and materials, University of Birmingham, Edgbaston, Birmingham, B15 2TT, United Kingdom.

* Correspondence: Czy805@gmail.com

Content

Text S1 Experimental Materials

Text S2 Preparation of $MoSe_2$

Text S3 Preparation of NH_2-MIL-101(Fe)

Text S4 Catalytic Degradation Experiment

Text S5 Analytical Methods

Table S1 Comparison of Removal Efficiency

Table S2 Changes in pH during the degradation of OFX at different initial pH values

Figure S1 Cycling stability experiments (a), (b) of the MSN-3/PMS system.

Figure S2 pH variation trend

Figure S3 Post-reaction XRD pattern

Figure S4 EPR spectrum under pH=11

Figure S5 Phenol removal rate (a); pH change of the solution (b) after reacting phenol with quenchers alone for 60 min.

Text S1 Experimental Materials

Reagents used in this study, including terephthalic acid, 2-aminoterephthalic acid, iron(III) chloride hexahydrate ($FeCl_3{\cdot}6H_2O$), N,N-dimethylformamide (DMF), and absolute ethanol, were all of analytical grade and purchased from Shanghai Aladdin Biochemical Technology Co., Ltd. Ammonium molybdate tetrahydrate and phenol were purchased from Shanghai Macklin Biochemical Technology Co., Ltd. Unless otherwise stated, all solutions were prepared using deionized water.

Text S2 Preparation of $MoSe_2$

First, 0.48 g (6 mmol) of selenium powder was added to 15 mL of hydrazine hydrate solvent, and after stirring for 30 min, a dark red-brown mixture A was obtained. Next, 0.72 g (3 mmol) of sodium molybdate dihydrate was added to 75 mL of deionized water, and after stirring for 30 min, a transparent mixture B was obtained. Then, mixture A was added to mixture B and stirred for 1 hour. After thorough mixing, the mixture was transferred to a PTFE-lined vessel and reacted at 200°C for 4 hours. After the reactor cooled to room temperature, it was washed multiple times with deionized water and anhydrous ethanol to obtain the black product MoSe2. The preparation process is shown in Fig.1.

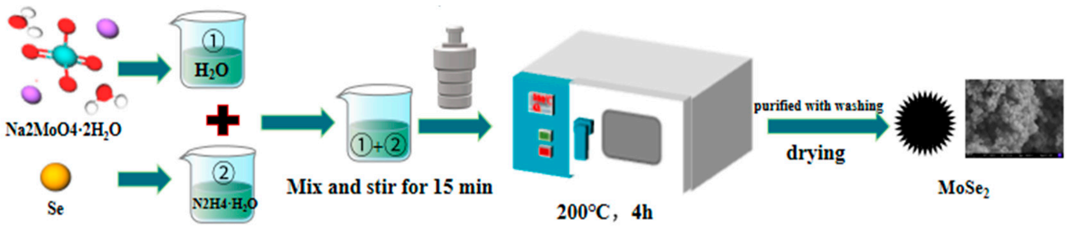

Figure 1. Flow chart of the preparation process of MoSe2

Text S3 Preparation of NH_2-MIL-101(Fe)

Fe(III) chloride hexahydrate ($FeCl_3$•6H_2O, 0.865 g, 3.2 mmol) and Fe(III) nitrate nonahydrate ($Fe(NO_3)_3$•9H_2O, 1.293 g, 3.2 mmol) were used as iron sources respectively. Each iron source was dissolved in 10 mL of DMF and stirred for 15 minutes under a magnetic stirrer to obtain Solution A. Subsequently, 0.290 g (1.6 mmol) of 2-aminoterephthalic acid (NH_2-BDC) was weighed and dissolved in 10 mL of DMF, followed by stirring for 15 minutes to prepare Solution B. Solutions A and B were mixed and then transferred into a stainless steel autoclave lined with polytetrafluoroethylene (PTFE). The autoclave was placed in an oven and reacted at 110 °C synthesis temperatures for 24 h synthesis durations . After naturally cooling to room temperature, the product was alternately washed with DMF and absolute ethanol several times to remove residual impurities. The washed material was then dried overnight in a vacuum drying oven at 70 °C to obtain the final product as a brown powder. For the preparation of MIL-101(Fe), 2-aminoterephthalic acid (NH_2-BDC) was replaced with terephthalic acid (H_2-BDC), while all other preparation procedures remained identical. The preparation flowchart is shown in Figure 2.

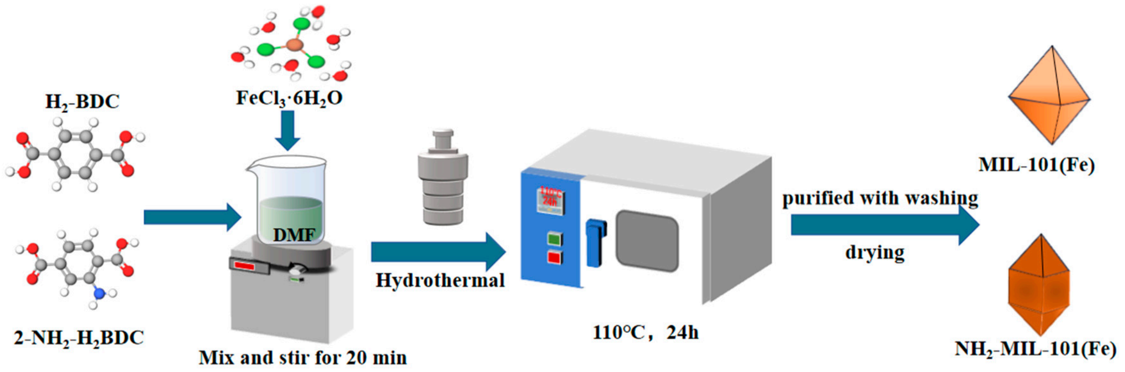

Figure 2. Flow chart of the preparation process of NH2-MIL-101(Fe)

Text S4 Catalytic Degradation Experiment

Phenol (Phenol) with an initial concentration of 20 mg/L was used as the target pollutant. Meanwhile, ofloxacin (OFX), norfloxacin (NOR), and rhodamine B (all with a concentration of 20 mg/L) were selected as representative typical organic pollutants to verify the universality of the catalyst.

The effects of different $MoSe_2$ doping ratios, MSN-3 dosages, PMS concentrations (0.25, 0.5, 1, 1.5, and 2 mmol/L), initial pH values, and reaction temperatures were systematically investigated. Meanwhile, the influence of common anions in water on the oxidative degradation process of phenol was analyzed. The degradation rate constant of phenol was calculated based on the pseudo-first-order kinetic model.

Furthermore, to systematically evaluate the cyclic performance of MSN-3, centrifugation was used to recover the catalyst after the reaction in this study. After the reaction, the recovered catalyst was alternately washed with N,N-dimethylformamide (DMF) and absolute ethanol 3 times (10 mL each time, shaking for 10 min) to completely remove phenol and reaction intermediates adsorbed on its surface. Subsequently, the catalyst was dried to a constant weight in a vacuum drying oven at 60 °C and reused for the next phenol degradation experiment; a total of 4 cyclic tests were completed.

Text S5 Analytical Methods

All experimental samples are grinded into powder prior to characterization. Scanning electron microscopy (SEM) and X-ray energy dispersive spectroscopy (EDS) are used to analyze the microstructural characteristics of the samples (Model JSM-5600 LV, JEOL, Japan). N_2 adsorption-desorption (BET, ASAP 2460, Micromeritics, USA) experiments are combined to determine the specific surface area and pore size distribution of the materials. X-ray diffraction (XRD, D/max-2400, Tokyo, Japan) analysis is employed to verify the crystal structure integrity of the MSN-3 composite. Fourier transform infrared spectroscopy (FT-IR, IRprestige-21, Tokyo, Japan) and X-ray photoelectron spectroscopy (XPS, EscaLab 250Xi, Waltham, MA, USA)) analyses are conducted to reveal the types of chemical functional groups on the sample surface and the chemical states of elements.

Table S1 Comparison of Removal Efficiency

Composite Materials	Dosage	PMS	Phenol	Reaction Time	Removal Rate	Remarks	References
$NiFe_2O_4$/HA	0.6 g/L	3 mmol/L	50 mg/L	60 min	98.50%		[1]
Fe-MOFs	0.7 g/L	3 mmol/L	50 mg/L	60 min	92.00%		[2]
NH_2-MIL-101 (Fe, Ce)	0.2 g/L	2 mmol/L	10 mg/L	50 min	100.00%	0.059 min^{-1}	[3]
MnOOH/g-C_3N_5	0.5 g/L	6 mmol/L	25 mg/L	10 min	99.90%		[4]
Co_2MnO_4	0.2 g/L	2.0 g/L	50 mg/L	45 min	100%	0.076 min^{-1}	[5]
Mn_3O_4/λ-MnO_2//PMS	0.2 g/L	0.8 g/L	20 mg/L	60 min	83.10%	0.034 min^{-1}	[6]
$MoSe_2$/NH_2-MIL-101(Fe)	0.3 g/L	7.5 mmol/L	20 mg/L	30 min	90.00%	0.073 min^{-1}	This study

Table S2 Changes in pH during the degradation of phenol at different initial pH values

Initial pH	3	5	7	9	11
pH value (with catalyst and PMS added)	2.78	3.55	3.61	3.88	6.92
pH value (after 60 minutes of reaction)	2.77	3.54	3.56	3.86	6.73

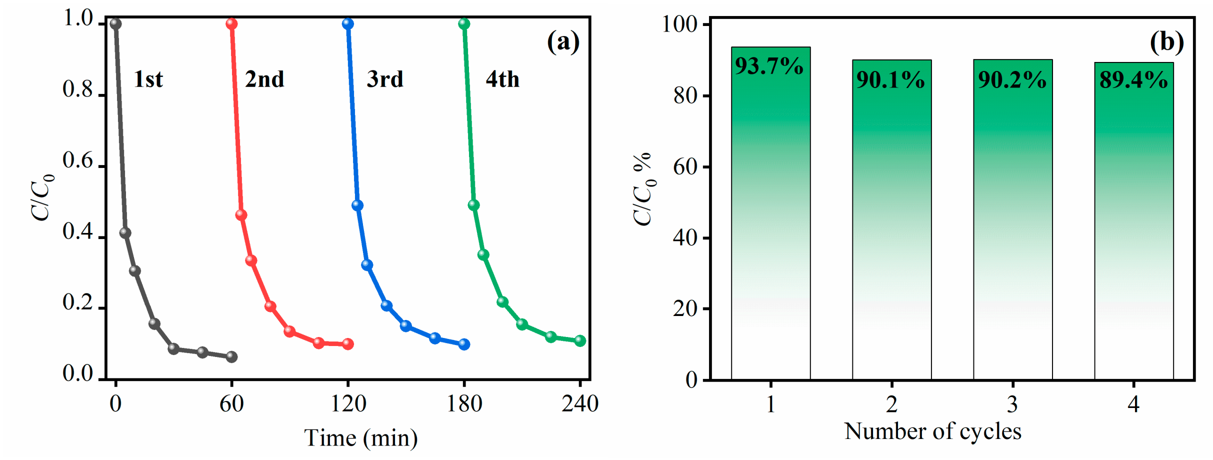

Figure S1 Cycling stability experiments (a), (b) of the MSN-3/PMS system.

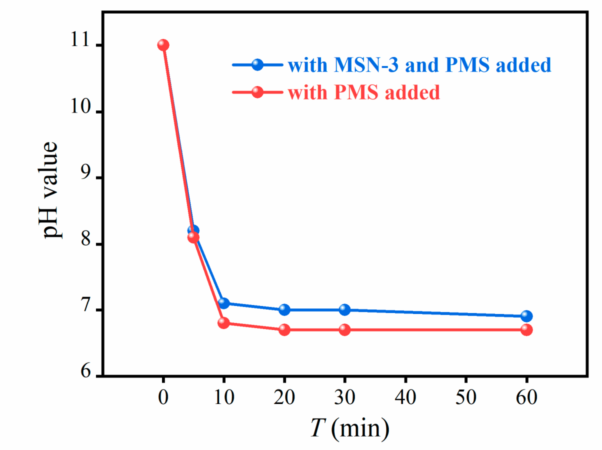

Figure S2 pH variation trend

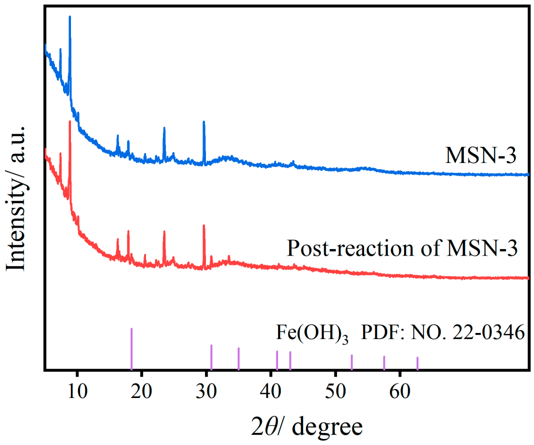

Figure S3 Post-reaction XRD pattern

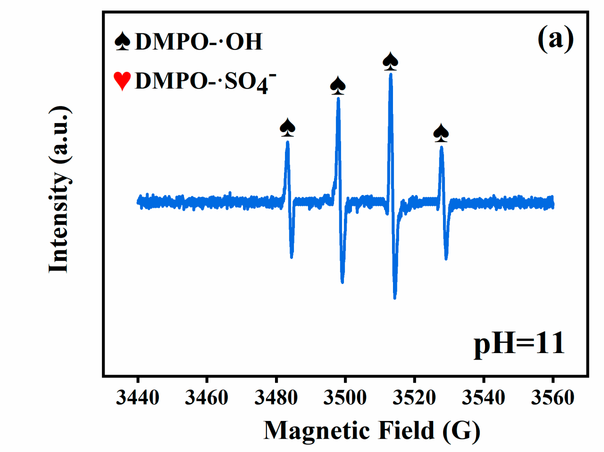

Figure S4 EPR spectrum under pH=11

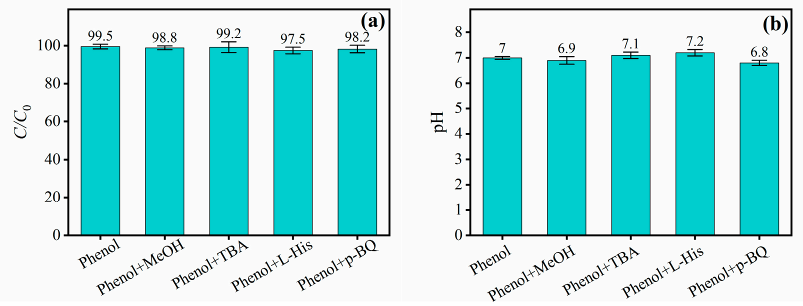

Figure S5 Phenol removal rate (a); pH change of the solution (b) after reacting phenol with quenchers alone for 60 min.

Experimental conditions: initial phenol concentration 20 mg/L, solution volume 200 mL, initial pH=7.0, temperature 25±2℃, only quenchers added (no MSN-3 catalyst, no PMS), reaction time 60 min.

References

[1] S. Wang, Y. Zhang, X. Huang, 2024. Hydroxylamine hydrochloride-driven activation of NiFe2O4 for the degradation of phenol via peroxymonosulfate. Environmental Research 263, 120057. https://doi.org/https://doi.org/10.1016/j.envres.2024.120057.

[2] S. Wang, Y. Dai, H. Wang, et al., 2024. Activation of peroxymonosulfate by Fe based metal organic framework for selective degradation of phenol in water. Journal of Water Process Engineering 68, 106454. https://doi.org/https://doi.org/10.1016/j.jwpe.2024.106454.

[3] L. Mo, G. Chen, B. Xu, 2024. Degradation of phenol by peroxymonosulfate catalyzed by cerium-doped amino-functionalized metal-organic frameworks (NH2-MIL-101 (Fe, Ce)). Journal of Environmental Chemical Engineering 12(4), 113256. https://doi.org/https://doi.org/10.1016/j.jece.2024.113256.

[4] X. Jiang, Y. Xing, X. Jin, et al., 2024. Activation of peroxymonosulfate by MnOOH/g-C3N5: Study on highly selective removal of phenolic pollutants and its non-radical pathway. Journal of Environmental Chemical Engineering 12(6), 114636. https://doi.org/https://doi.org/10.1016/j.jece.2024.114636.

[5] L. Yue, L. Hao, J. Zhang, et al., 2023. Oxygen-enriched vacancy Co2MnO4 spinel catalyst activated peroxymonosulfate for degradation of phenol: Non-radical dominated reaction pathway. Journal of Water Process Engineering 53, 103807. https://doi.org/https://doi.org/10.1016/j.jwpe.2023.103807.

[6] Z. Xing, M. Fan, J. Liu, et al., 2023. A novel Fenton-like catalyst and peroxymonosulfate activator of Mn3O4/λ-MnO2 for phenol degradation: Synergistic effect and mechanism. Inorganic Chemistry Communications 150, 110396. https://doi.org/https://doi.org/10.1016/j.inoche.2023.110396.
